# Unleashing the Power of Cold Atmospheric Plasma: Inducing Mitochondria Damage‐Mediated Mitotic Catastrophe

**DOI:** 10.1002/advs.202401842

**Published:** 2024-10-23

**Authors:** Shengjie Peng, Yue Feng, K.N. Yu, Lijun Wu, Guodong Chen, Miaomiao Yang, Lele Zhao, Wei Cao, Qianwen Cui, Lianjun Chen, Quan Li, Yifan Huang, Cheng Cheng, Fengqin Zhu, Wei Han

**Affiliations:** ^1^ Anhui Province Key Laboratory of Medical Physics and Technology Institute of Health and Medical Technology Hefei Institutes of Physical Science Chinese Academy of Sciences Hefei 230031 P. R. China; ^2^ University of Science and Technology of China Hefei 230026 P. R. China; ^3^ Teaching and Research Section of Nuclear Medicine School of Basic Medical Sciences Anhui Medical University Hefei 230032 P. R. China; ^4^ Department of Physics City University of Hong Kong Tat Chee Avenue Kowloon Tong Hong Kong P. R. China; ^5^ State Key Laboratory in Marine Pollution City University of Hong Kong Tat Chee Avenue Kowloon Tong Hong Kong P. R. China; ^6^ Institute of Physical Science and Information Technology Anhui University Hefei 230031 P. R. China; ^7^ Hefei Cancer Hospital Chinese Academy of Sciences Hefei 230031 P. R. China; ^8^ School of Biology Food and Environment Hefei University Hefei 230601 P. R. China; ^9^ Department of Radiation Medicine School of Public Health and Management Wenzhou Medical University Wenzhou 325035 P. R. China; ^10^ Institute of Plasma Physics Hefei Institutes of Physical Science Chinese Academy of Sciences Hefei 230031 P. R. China; ^11^ Collaborative Innovation Center of Radiation Medicine of Jiangsu Higher Education Institutions and School for Radiological and Interdisciplinary Sciences (RAD‐X) Soochow University Suzhou 215006 P. R. China

**Keywords:** anti‐tumor effect, cold atmospheric plasma, mitochondria damage, mitotic catastrophe

## Abstract

Despite the promise of cold atmospheric plasma (CAP) for cancer treatment, the challenges associated with the treatment of solid tumors and penetration depth limitations remain, restricting its clinical application. Here, biological evidence is provided that the killing effect of CAP treatment is confined to less than 500 µm subcutaneously and the actual biological dose decreased gradually with depth for the first time, indicating that the limited penetration depth has become an urgent problem that demands immediate solutions. Significantly, it is showed that different from high‐dose treatments, CAP decreased the doses to the low‐dose range but still exhibited anti‐tumor effects via mitotic catastrophe. Unlike radiotherapy or chemotherapy, low‐dose CAP treatment induces mitochondrial structural damage and dysfunction, disrupts energy metabolism and redox balance, and results in mitotic catastrophe. Collectively, these findings suggest that better understanding and taking full advantage of the dose‐response gradient effect of CAP is a potential strategy to prompt its clinical application beyond improving CAP penetration.

## Graphical abstract

This study focuses on the limited cold atmospheric plasma (CAP) penetration, a major challenge for its clinical application, in vivo and the results show the depth‐dependent biological effect of CAP treatment for the first time. Mitochondria damage and dysfunction induced by low dose CAP suppress microtubule assemble and spindle bipolar, and ultimately result in mitotic catastrophe to suppress tumor growth.

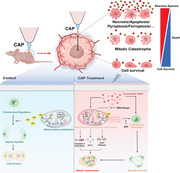

## 1 Introduction

Plasma, the fourth state of matter (in addition to the solid, liquid, and gas states), is an ionized gas composed of ions, electrons, neutral species radicals, and photons. Cold atmospheric plasma (CAP) is a type of plasma produced at low‐ambient temperatures, and its biological safety has been proven in numerous studies.^[^ ^1^ ^]^ To date, CAP has been reported to exert a notable killing effect in various types of cancer cells, relying mainly on the synergistic effect of induced reactive oxygen species (ROS) and reactive nitrogen species (RNS) in targeted cells and tissues, ultimately inducing multiple types of programmed cell death, including apoptosis, autophagy, senescence, and pyroptosis.^[^ ^2^, ^3^, ^4^, ^5^ ^]^ While the valuable anti‐tumor capacity has prompted studies on CAP cancer therapies,^[^ ^6^ ^]^ one of the major challenges to clinical applications is the poor penetration of the active components into the treated tissues.^[^ ^7^ ^]^ The physical penetration of CAP is estimated to be only a few hundred micrometers, which limits its biological effects by the persistent attenuation of plasma energy and deep reactive species penetration into the deeper tissues.^[^ ^8^, ^9^, ^10^ ^]^ Hence, the efficacy of CAP remains unsatisfactory, and frequent CAP treatment may be required to achieve the desired therapeutic effects.

Mitotic catastrophe, initially identified and described as a lethal phenotype in yeast strains in the 1980s,^[^ ^11^ ^]^ is regarded as an intrinsic oncosuppressive mechanism that prevents cell survival through incomplete mitosis owing to defects in the mitotic apparatus, DNA damage, and mitotic checkpoint errors.^[^ ^11^, ^12^, ^13^ ^]^ Functionally, the induction of mitotic catastrophe, which can suppress tumorigenesis and cancer progression, appears to be a promising strategy for cancer treatment.^[^ ^14^ ^]^ Importantly, mitotic catastrophe contributes to the induction of polyploid and aneuploid cells, which suffer from various cellular stresses, including elevated ROS levels, and exhibit increased sensitivity to therapeutics.^[^ ^15^, ^16^ ^]^ Notably, mitotic catastrophe is one of the crucial mechanisms for radiotherapy, chemotherapy pharmaceuticals such as paclitaxel, doxorubicin, camptothecin^[^ ^17^, ^18^, ^19^ ^]^ and other therapies that have anti‐tumor effects. Mechanistically, tumor cells are highly sensitive to the induction of mitotic catastrophe due to genomic instability, which makes them more prone to mitotic aberrations,^[^ ^20^ ^]^ suggesting that the induction of mitotic catastrophe might be an attractive strategy for the development of novel anti‐tumor therapies. 

In this study, the relationship between the biological effects and penetration depth of CAP in tumor xenografts was quantified for the first time. Furthermore, we revealed that relatively low‐dose CAP treatment inhibited the proliferation of various tumor cells and distinctly induced accumulative mitotic catastrophe owing to the destructive effect of CAP treatment on microtubule dynamics and centrosome maturation. The study revealed that CAP‐derived ROS resulted in severe damage to mitochondrial structure and dysfunction, increasing mitochondrial ROS levels, and energy metabolism homeostasis disequilibrium, eventually inducing mitotic catastrophe. We hope that our work can promote a better understanding of CAP treatment of tumors, which may provide new insights into its clinical application and unveil more therapeutic strategies.

## 2 Results

### 2.1 The Biological Effects of CAP Treatment Decrease with Depth in Tumor

Multiple studies have suggested that one of the major challenges in the clinical application of CAP is the poor penetration of active components into the treated tissues.^[^ ^7^ ^]^ The physical penetration of CAP is estimated to be only a few hundred micrometers, which limits its biological effects owing to the attenuation of plasma energy and decreased penetration of reactive species with increasing depth.^[^ ^8^, ^10^ ^]^

**Figure 1:**
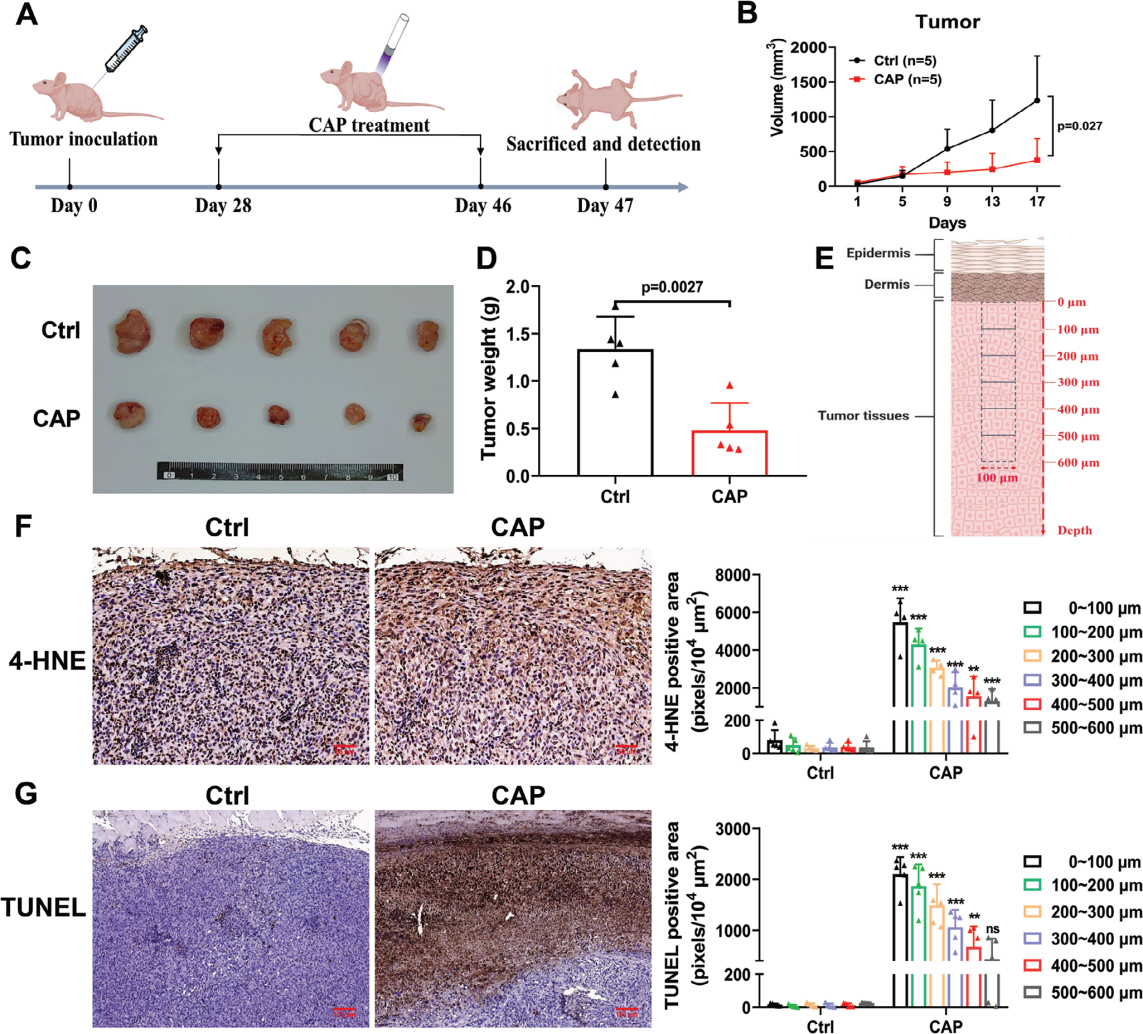
The biological effects of CAP treatment decrease with depth in tumor. A) Schematic of the jet plasma treatment on the xenograft tumor model in nude mice. B) Tumor growth curve. C) The images of excised tumors. D) The weight of excised tumors. E) The diagram for quantifying the biological effects of CAP in tumor tissues. The 4‐HNE and TUNEL signals per unit area (100 × 100 µm^2^) were quantified from 0 to 600 µm under the dermis. Three units were chosen randomly in each layer. F) The representative IHC images of 4‐HNE and the quantified result. Scale bar, 50 µm. G) The representative images of TUNEL and the quantified result. Scale bar, 100 µm. ** *p *< 0.005, *** *p *< 0.0001.

As shown in **Figure** **1**A, we established a tumor xenograft model (Calu‐1 cells) in nude mice and treated them with CAP (15 min every two days) for 20 days (Figure 1A). Both tumor volume and weight were significantly lower in the CAP‐treated group than in the control group (Figure 1B–D), indicating that CAP treatment effectively suppressed tumor growth in vivo. Moreover, we detected markers related to oxidative stress and cell death in subcutaneous tumors using immunohistochemistry (IHC). The levels of 4‐hydroxynonenal (4‐HNE) and terminal deoxynucleotidyl transferase‐mediated dUTP nick end labeling (TUNEL) positive cells in the treated group were both much higher than those in the control group, indicating that plasma treatment induced significant oxidative stress and cell death in tumor tissues (Figure 1F,G).

Furthermore, 4‐HNE and TUNEL positive cells in each tumor tissue layer (100 µm) were quantified (0–600 µm under the dermis layer) (Figure 1E). The biological effects (4‐HNE and TUNEL) induced by CAP treatment peaked in the surface layer (0 – 100 µm) of subcutaneous tumors, and then gradually declined with increasing depth (Figure 1F,G). These results confirm the limited depth of the biological effects of CAP in tissues, which is consistent with previous studies.^[^ ^7^ ^]^ Indeed, the penetration or diffusion of CAP components, especially plasma‐derived ROS/RNS, weakens rapidly with increasing tissue depth, which also means that the actual dose of CAP, quantified by its biological effects, progressively decreases with tissues depth.

### 2.2 CAP in Low Dose Range Inhibited Tumor Cell Growth

**Figure 2:**
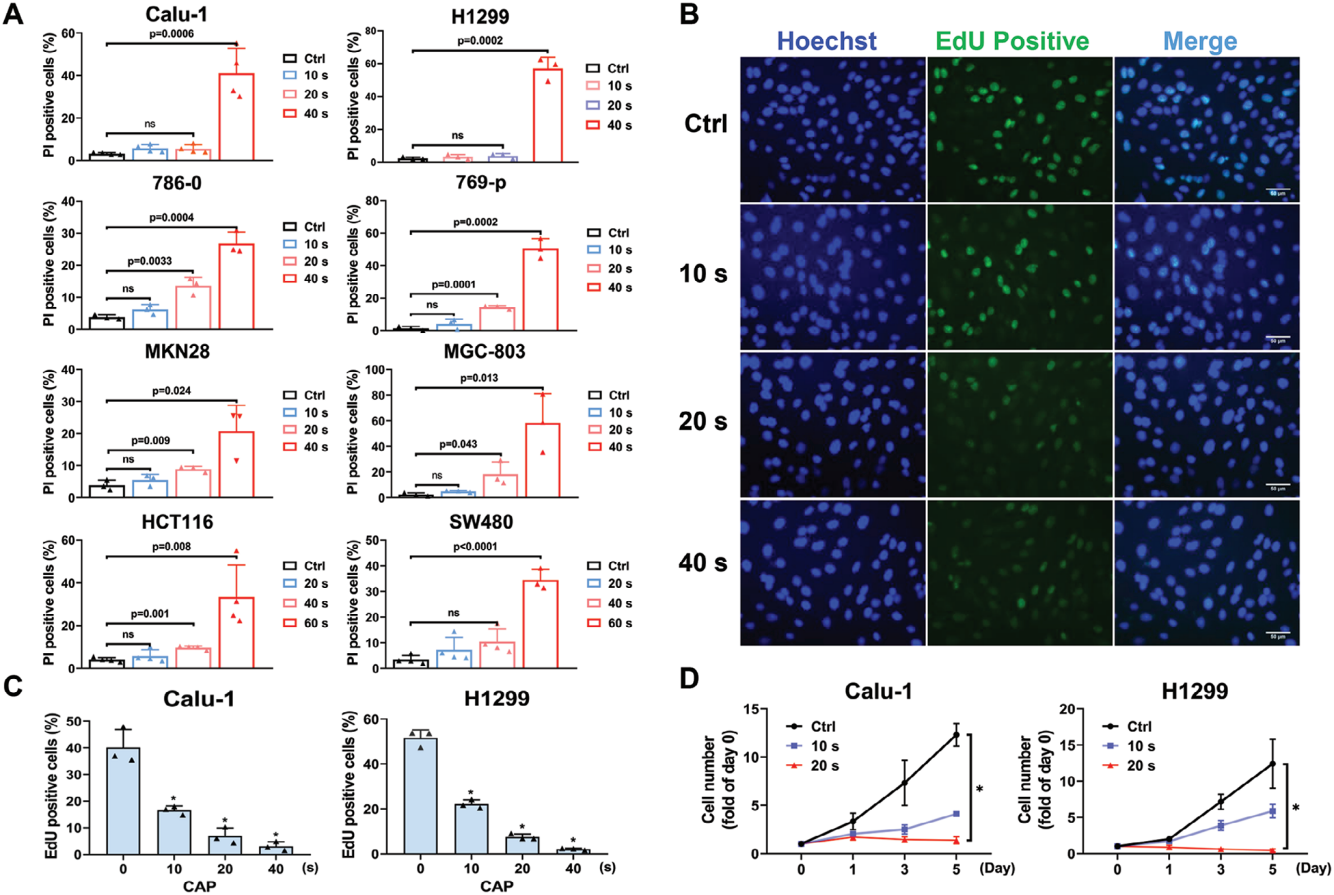
The low dose CAP inhibiting tumor cells proliferation. A) The cell death of eight types of cancer cell lines, treated with CAP (10 – 60 s), was detected with PI (5 µg mL^−1^) staining and analyzed with flow cytometry after 24 h. B) Typical images of proliferating cells in Calu‐1 cells labeled with EdU. Scale bar, 50 µm. C) Quantification of B. D) Cell number of Calu‐1/H1299 cells counted at indicated timepoints after CAP treatment (20 s) compared with control. * *p *< 0.001.

Regarding biological effects, the in vivo results showed that high‐dose CAP induced severe cell death in tumor cells, which is consistent with previous studies.^[^ ^21^, ^22^ ^]^ Whether CAP in the low‐dose range contributes to tumor suppression, although direct cell death was probably not induced, has not yet been explored. The potential role of low‐dose CAP in tumor control was studied in this study. We first investigated the relationship between the CAP dose and its cytotoxic effects on tumor cells. Cell death was detected using propidium iodide (PI) staining in lung cancer, gastric cancer, colon cancer, and renal carcinoma cell lines after CAP treatment. As shown in **Figure** **2**A, high‐dose CAP treatment (40 or 60 s) quickly induced cell death (24 h after treatment), whereas low‐dose treatment (10 or 20 s) did not show any distinct effects, indicating that the killing effect of CAP depended on the dose.

Although low‐dose CAP did not kill tumor cells significantly, the proliferation, assessed by the Click‐EdU assay, of tumor cells treated by low‐dose CAP (10 or 20 s) nonetheless decreased obviously (Figure 2B,C), indicating that cell proliferation has been distinctly suppressed. Moreover, we continuously counted the number of Calu‐1 and H1299 cells for five days after low‐dose treatment (10 or 20 s). The results showed that the number of cells in the control group increased rapidly, whereas those in the treated group remained at low levels, which revealed that low‐dose CAP treatment resulted in the inhibition of tumor cell growth (Figure 2D). It was intriguing to determine how low‐dose CAP treatment suppressed cell proliferation but did not promptly induce cell death.

### 2.3 Low Dose CAP Treatment Induced Mitotic Catastrophe via G2/M Arrest

**Figure 3:**
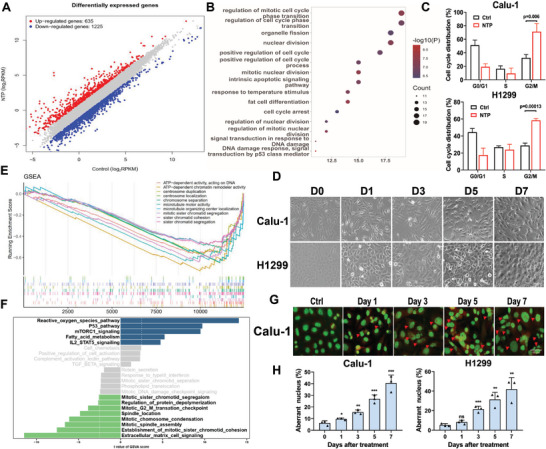
Low dose CAP treatment induced mitotic catastrophe via G2/M arrest. A) The differentially expressed genes enriched in treated Calu‐1 cells. B) Results of GO enrichment analysis. C) Cell cycle of Calu‐1 and H1299 cells detected at 48 h after CAP treatment. D) Morphology of treated Calu‐1 and H1299 cells captured at indicated timepoints. Scale bar, 100 µm. E) The mitosis related genes and pathways enriched by the GSEA analysis. F) The upregulated and downregulated pathways enriched by GSVA analysis. G) Nucleus morphology of treated Calu‐1 cells captured at indicated timepoints. Scale bar, 50 µm. H) Frequency of cells with aberrant nuclei. * *p *< 0.05, ** *p *< 0.005, *** *p *< 0.0001.

To further determine the mechanism underlying the effect of low‐dose CAP treatment on tumor cell growth, Calu‐1 cells were treated and collected for RNAseq analysis. The results of differentially expressed genes analysis showed that 635 genes were up‐regulated while 1225 genes were down‐regulated after CAP treatment (20 s) (**Figure** **3**A), in which the regulation of cell cycle and mitosis‐related biological processes were significantly enriched in gene ontology (GO) analysis (Figure 3B). For further verification, the cell cycle was analyzed using PI staining and flow cytometry. The results showed that the cell cycle of both Calu‐1 and H1299 cells was arrested at the G2/M phase after 20 s of CAP treatment (Figure 3C). Significant morphological changes, namely, enlarged or elongated sizes compared to the controls, were observed in CAP‐treated Calu‐1 and H1299 cells (Figure 3D), indicating the occurrence of accumulative abnormal mitosis.

To better the changes in associated signaling pathways, gene set enrichment analysis (GSEA) was applied and the results revealed that mitosis‐related pathways were all down‐regulated, including mitotic sister chromatid cohesion and segregation, centrosome duplication and location, and microtubule organization and localization (Figure 3E), and the gene set variation analysis (GSVA) also showed that the inhibition of mitosis‐related gene expression was associated with mitotic G2/M transition, spindle assembly, and chromosome condensation (Figure 3F). Furthermore, we identified the nuclear morphology with acridine orange staining and observed multinucleation and micronucleation after CAP treatment, which indicated the occurrence of mitotic catastrophe (Figure 3G, Extended Figure 1A). The number of cells with aberrant nuclei, including multi‐ and micronuclei, increased significantly in a time‐dependent manner after 20 s of CAP treatment (Figure 3H), indicating that low‐dose CAP treatment induced persist and wide‐range mitotic catastrophe in tumor cells.

### 2.4 CAP Treatment Suppressed Microtubule Dynamics and Centrosome Maturation

**Figure 4:**
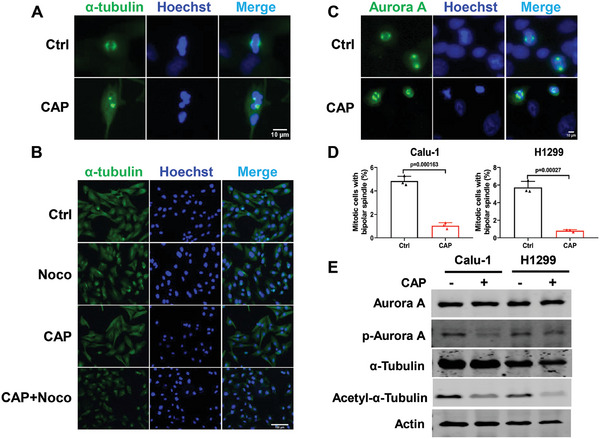
CAP treatment suppressed microtubule dynamics and centrosome maturation. A) Representative immunofluorescent images of α‐tubulin in Calu‐1 cells at 24 h after CAP treatment. Scale bar, 10 µm. B) Representative immunofluorescent images of α‐tubulin network in Calu‐1 cells at 24 h after CAP treatment with or without Nocodazole (5 µm) treatment. Scale bar, 100 µm. C) Representative immunofluorescent images of Aurora A in Calu‐1 cells at 24 h after CAP treatment. Scale bar, 10 µm. D) Frequency of mitotic cells with bipolar spindles. E) The expression of related proteins in Calu‐1 and H1299 at 24 h after CAP treatment.

The dynamic behavior of microtubules, a key component of the cytoskeleton, facilitates the positioning and stabilization of mitotic spindles as well as the search for and capture of mitotic chromosomes, while bipolar mitotic spindles carry out equal segregation of sister chromatids and cell division.^[^ ^23^, ^24^ ^]^ Based on the results of RNAseq analysis, we further detected microtubule formation and centrosome maturation, and the results showed that the cells treated with CAP (20 s) formed a disordered structure with monopolar or multipolar microtubules, labeled by the immunofluorescence of α‐tubulin, while the control cells still showed well‐organized microtubule networks with a bipolar array of microtubules (**Figure** **4**A). Furthermore, Calu‐1 cells treated with CAP were incubated with nocodazole, a rapid reversible inhibitor of microtubule, and microtubule regrowth was immediately observed. The cells treated with CAP displayed moderate microtubule regrowth compared with the control cells (Figure 4B). In addition, we detected the protein expression of α‐tubulin and noticed that the acetylation level of α‐tubulin decreased after treatment (Figure 4E), indicating that CAP treatment inhibited microtubule assembly and polymerization.

The fluorescence of Aurora A, a widespread protein kinase localized at mitotic centromeres and spindles that plays key roles during cell division, showed deranged centrosomes with multipolar or monopolar (Figure 4C,D), which was consistent with the above results in the treated cells. Furthermore, the level of phosphorylated Aurora A decreased (Figure 4E), indicating that CAP treatment suppressed the maturation and replication of centrosomes and induced mitotic catastrophe in vitro.

### 2.5 CAP Treatment Induced Mitochondrial Damage

**Figure 5:**
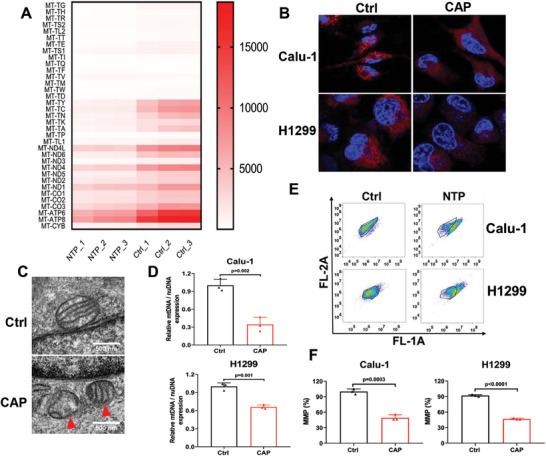
CAP treatment (20 s) induced mitochondrial damage. A) The mitochondria‐related biological processes enriched by GO analysis. B) Representative immunofluorescent images of mitochondria (red) and nucleus (blue) in Calu‐1 and H1299 cells with confocal microscopy at 24 h after CAP treatment. C) Representative images of mitochondria in Calu‐1 cells with transmission electron microscopy at 24 h after CAP treatment. Scale bar, 500 nm. D) The ratio of mtDNA to nucleus DNA. E) The mitochondrial membrane potential after CAP treatment detected with flow cytometry. F) Quantification of E.

The results of RNAseq analysis revealed that the expression levels of almost all mitochondria‐related genes were decreased, including tRNA and mitochondrial structural proteins (**Figure** **5**A), suggesting potential damage to mitochondria after CAP treatment. In addition, we observed morphological changes in the mitochondria stained with the MitoTracker probe in Calu‐1 cells using confocal microscopy. Decreased distribution and abnormal shape of the mitochondria were observed after treatment, indicating that CAP might damage the mitochondria (Figure 5B). Transmission electron microscopy images also showed diminished or disappeared cristae in the mitochondria (Figure 5C), indicating that CAP treatment significantly damaged the mitochondrial structure. Furthermore, to verify the changes in mitochondrial biogenesis and mass, the ratio of mitochondrial DNA (mtDNA) to nuclear DNA was assessed via qPCR. The significantly decreased ratio (Figure 5D) suggested that mitochondrial biogenesis was distinctly suppressed after CAP treatment. Additionally, significantly reduced membrane potential and mitochondrial depolarization indicated a decline in mitochondrial integrity after CAP treatment (Figure 5E,F).

### 2.6 CAP Treatment Induced Mitochondrial Dysfunction

**Figure 6:**
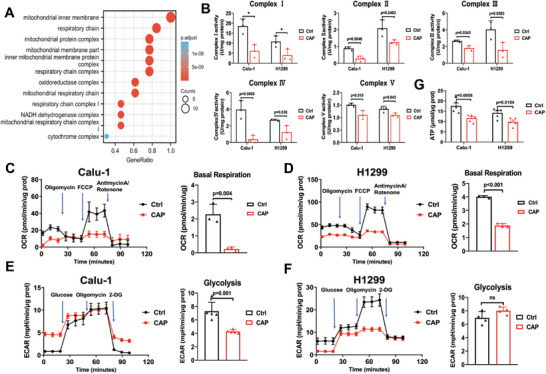
CAP treatment (20 s) induced mitochondrial dysfunction. A) The mitochondria related components enriched by GO analysis. B) The activity of mitochondrial respiratory complex I – V detected at 24 h after CAP treatment. C) OCR and the basal respiratory level of Calu‐1 cells after CAP treatment. D) OCR and the basal respiratory level of H1299 cells after CAP treatment. E) ECAR and the glycolysis level of Calu‐1 cells after CAP treatment. F) ECAR and the glycolysis level of H1299 cells after CAP treatment. G) The ATP level in Calu‐1 and H1299 cells after CAP treatment.

The results of GO enrichment analysis showed that multiple mitochondrial biological processes were considerably affected, including the respiratory chain complex and oxidoreductase complex (**Figure** **6**A). Further investigation confirmed that the activities of all complexes (I – V) decreased distinctly in Calu‐1 and H1299 cells after CAP treatment (Figure 6B). Subsequently, the mitochondrial metabolism was assessed with a Seahorse XFe96 Bioenergetic Flux Analyzer. The results showed that the mitochondrial O_2_ consumption rate (OCR) in treated cells was obviously decreased, with a notable reduction in basal respiration and ATP production compared to the control level (Figure 5C,D, and Extended Figure 1B,C), indicating a decreased level of mitochondrial respiration. Meanwhile, the extracellular acidification rate (ECAR) was also significantly reduced along with attenuated glycolytic capacity and reserve (Figure 6E,F, and Extended Figure 1D,E), indicating the decline in glycolysis.

To further verify the dysfunction of mitochondrial energy metabolism, the intracellular ATP levels were measured. The results showed that ATP levels were considerably reduced after CAP treatment (Figure 6G). In response to the ATP levels decline, activation of the AMPK pathway, shown as an increased level of phosphorylated AMPK, also indicated the inadequacy of ATP supply and the destruction of energy metabolism homeostasis (Extended Figure 1F).

### 2.7 Mitochondrial Dysfunction Elicited Mitotic Catastrophe after CAP Treatment

Based on GSEA analysis, ATP‐dependent chromatin remodeling activity and ATP‐dependent activity were both down‐regulated (Figure 3E). CAP treatment also decreased ATP production (Figure 6G). We hypothesized that ATP deficiency and excessive ROS levels may contribute to mitotic catastrophe.

**Figure 7:**
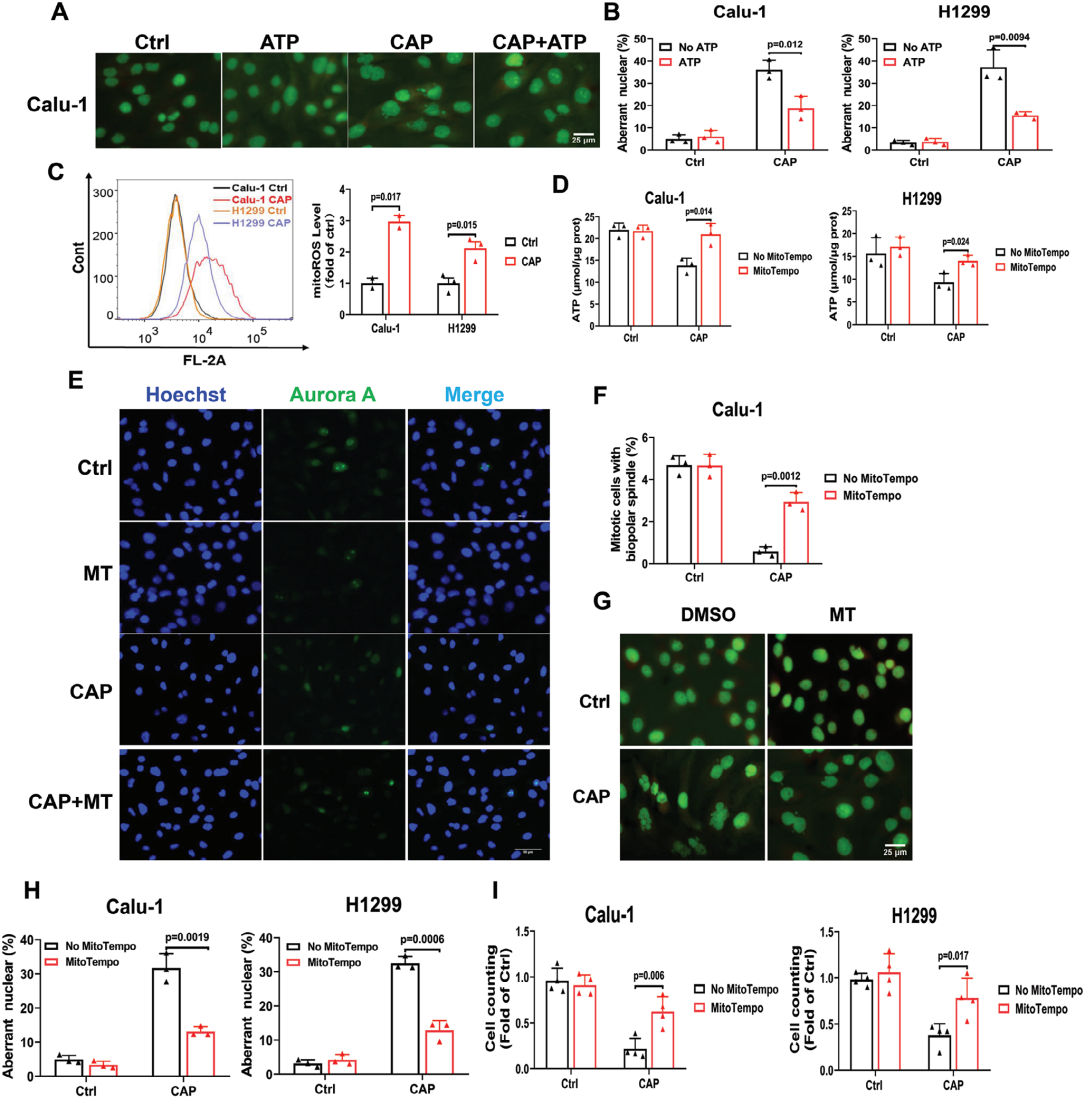
Mitochondrial dysfunction elicited mitotic catastrophe after CAP treatment (20 s). A) Representative immunofluorescent images of nuclei (green) in Calu‐1 cells at 72 h after CAP treatment with or without ATP (100 µm). Scale bar, 25 µm. B) Quantified results of A. C) The mitoROS level detected with MitoSOX Red after CAP treatment. D) The ATP level in Calu‐1 and H1299 cells after CAP treatment with or without MitoTempo (300 µm). E) Representative immunofluorescent images of Aurora A in Calu‐1 cells at 72 h after CAP treatment with or without MitoTempo (300 µm). Scale bar, 50 µm. F) Quantified result of E. G) Representative immunofluorescent images of nuclei in Calu‐1 cells at 72 h after CAP treatment with or without MitoTempo (300 µM). Scale bar, 25 µm. H) Quantified result of G. I) The number of cells at 72 h after CAP treatment with or without MitoTempo (300 µm).

We supplemented the culture medium with exogenous ATP (100 µm) before CAP treatment to rescue the decline in endogenic ATP levels and then examined the occurrence of multinucleation and/or micronucleation. ATP treatment significantly decreased the frequency of aberrant nuclei (multinucleation and/or micronucleation) (**Figure** **7**A,B, Extended Figure 2A) and promoted the proliferation of CAP‐treated Calu‐1 and H1299 cells (Extended Figure 2B).

Considering that mitochondria are the main source of intracellular ROS production to induce oxidative stress in cells, we detected the generation of mitochondria ROS (mitoROS) with its specific probe, MitoSOX Red. As shown in Figure 7C, CAP treatment induced a distinct production of mitoROS. Moreover, mitoROS scavenging with MitoTempo effectively rescued the decline in ATP levels in Calu‐1 and H1299 cells after CAP treatment (Figure 7D). Furthermore, MitoTempo treatment significantly decreased the frequency of aberrant spindles in CAP‐treated cells (Figure 7E,F). In addition, the frequency of aberrant nuclei after CAP exposure was effectively suppressed by treatment with MitoTempo (Figure 7G,H), which finally resulted in the restoration of cell proliferation of Calu‐1 and H1299 cells from the growth inhibition induced by CAP treatment (Figure 7I).

### 2.8 Low‐Dose CAP Treatment Induced Mitotic Catastrophe In Vivo

**Figure 8:**
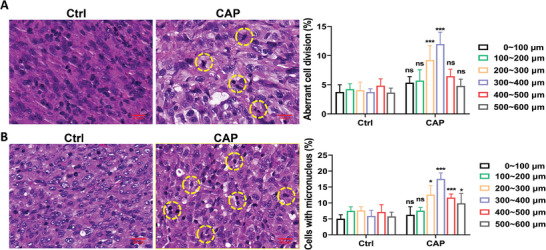
CAP induced abnormal mitosis in vivo. A) The representative H&E images of aberrant cell division and the quantified results. Scale bar, 20 µm. B) The representative H&E images of micronuclei and the quantified results. Scale bar, 20 µm; *p < 0.05, **p < 0.005, ***p < 0.0001.

In the xenograft model in nude mice treated with CAP described above (Figure 1A), the level of aberrant nuclei and micronuclei in tumor tissues increased distinctly based on hematoxylin and eosin (H&E) staining, which showed the occurrence of abnormal nuclear division under the layers of the cortex, especially at 300 – 400 µm (**Figure** **8**A,B). Different from the decreasing pattern of TUNEL and 4‐HNE positive signals with increasing depth, the frequency of abnormal nuclear division was not higher than that of the control in the superficial layers (0 – 200 µm) of subcutaneous tumors, where the actual dose of CAP was relatively high. The frequency reached a peak in the 300 – 400 µm layer, where the dose of CAP had already gradually declined. Together with the results of previous studies,^[^ ^25^ ^]^ it is considered that low‐dose CAP can suppress tumor growth by inducing mitotic catastrophe rather than inducing other types of programmed cell death directly in the tumor cells. This result also suggests the complexity of cell death types in CAP‐treated tumors and the possible switching of different cell death types over a wide range of CAP dose.

Taken together, these results indicate that microtubule dynamics and centrosome maturation are suppressed by CAP treatment, contributing to mitotic catastrophe in vitro and in vivo.

## 3 Discussion

Solid tumors have a 3D structure and a defined volume in vivo. One of the current challenges in the clinical application of CAP is its penetration in tissues. Until now, numerous studies have explored the penetration and diffusion of CAP‐generated radicals and have suggested that plasma penetration is estimated to be only a few hundred micrometers for the blocking effect of the cortex^[^ ^26^ ^]^ and limits the biological effects of reactive radicals.^[^ ^7^ ^]^ In order to study the penetration effect of CAP, scientists have constructed numerous experimental research models. Chen et al. developed a model framework for plasma‐biofilm and plasma‐tissue interactions to explore the plasma penetration, and the results showed that penetration of plasma into highly hydrated biofilms was about 10 – 20 µm.^[^ ^27^ ^]^ Nie et al. used the ex vivo tissue model to study the penetration of long‐life ROS/RNS generated by CAP and the results revealed that the serum concentration, the thickness of the tissue and other factors significantly affected the penetration of CAP.^[^ ^28^ ^]^ Wenzel et al. employed noninvasive and marker‐independent Raman microspectroscopy to assess the changes of DNA and lipids in primary human cervical tissue samples after CAP treatment and showed that the average CAP penetration depth was roughly 270 µm.^[^ ^29^ ^]^ However, these methods still only can be carried out in isolated tissues, and it remains impossible to monitor the radicals in real time in the in vivo tissues. Since the biological effects were induced by the active ingredients of CAP, we detected the biological effects of CAP with depth as an alternative method for directly checking the process of penetration and attenuation of plasma‐generated active ingredients in tissues. Herein, we first presented direct biological evidences that the biological effect of CAP treatment was confined to less than 500 µm subcutaneously (Figure 1F,G), and that the dose deposited in the deeper tissue layers would decrease into the low‐dose range due to the decreased levels of radicals.

For the limited penetration and diffusion of CAP‐generated radicals in vivo, CAP efficacy remains unsatisfactory and multiple and frequent CAP treatments are required to achieve clinically desirable therapeutic effects. New strategies are available to alleviate the penetration limitations and to promote CAP application. Chen et al. designed a hollow‐structured microneedle patch as microchannels to allow CAP to pass through the skin into tumors and cause tumor cell death.^[^ ^26^ ^]^ Coalescing the CAP technology with nanotechnology or chemotherapeutic drugs is another prospective approach, including magnetic nanoparticles,^[^ ^30^ ^]^ lipidic nanoparticles,^[^ ^31^ ^]^ temozolomide,^[^ ^32^ ^]^ and cisplatin.^[^ ^33^ ^]^ In our study, we discovered that low‐dose CAP treatment also exhibited tumor suppression effects by disrupting mitotic processes (Figures 2 and 3). In addition, polyploid and aneuploid tumor cells induced by mitotic catastrophe suffer from serious cellular stress and exhibit high sensitivity to therapeutics. Hence, combining CAP with other clinical treatments, including chemoradiotherapy, photodynamic therapy and nanoparticles, is a promising strategy.

Multiple types of CAP‐induced programmed cell death have been reported, including pyroptosis, ferroptosis, necroptosis, and autophagy.^[^ ^25^, ^34^ ^]^ As a novel cell death mode, mitotic catastrophe has attracted much attention because it is an intrinsic onco‐suppressive mechanism, and the induction of mitotic catastrophe may be an attractive target for the development of novel anti‐tumor therapies.^[^ ^12^ ^]^ In this study, we focused on the effect of low‐dose CAP on mitosis progression and found a disordered microtubule network and disturbed mitotic spindle formation (Figure 4A,C). Hara et al. verified that CAP treatment significantly activated the ATM/P53 pathway and blocked the cell cycle at the G2/M phase in lung cancer cells.^[^ ^35^ ^]^ Javier et al. also confirmed that CAP blocked the progression of mitosis via promoting phosphorylation of both CHK1 and p53 in human cholangiocarcinoma cells.^[^ ^36^ ^]^ Furthermore, we confirmed that the increasing mitoROS levels and ATP deficiency caused by the damage and dysfunction of mitochondria induced by CAP treatment resulted in the inhibition of microtubule dynamics, spindle assembly and eventually the induction of accumulative mitotic catastrophe in wide‐range tumor cells (Figure 7).

Among organelles injuries induced by CAP treatment, mitochondrial damage has been previously studied. Mitochondria are important sites of cell metabolism and sensitive to oxidative stress for naked mtDNA and high ROS production.^[^ ^37^ ^]^ Previous studies have showed that CAP treatment led to enhanced generation of intracellular ROS, decreased mitochondrial membrane potential, and induction of apoptosis through the release of cytochrome *c* and activation of the MAPK pathway in head and neck cancer cells.^[^ ^38^ ^]^ Panngom et al. discovered that CAP significantly reduced mitochondrial enzyme activity, respiration rate, and ATP production in lung cancer cells.^[^ ^39^ ^]^ Although the mitochondrial damage caused by CAP has been investigated in many studies, the specific targets and mechanisms of damage remain unclear. In our study, we found that CAP affected mitochondrial structure and distribution, inhibited the replication and reduced membrane potential (Figure 5B–F), which is in accordance with previous studies. We also revealed that the expression of almost all mitochondrial genes was down‐regulated, and the activities of all respiratory chain complexes decreased after CAP treatment, resulting in unbalanced energy metabolism and increased mitoROS generation (Figure 6). In addition to microtubule and spindle dysfunction, caspase 2 activation,^[^ ^40^ ^]^ cytochrome *c* release,^[^ ^41^ ^]^ and mitoCa^2+^ oscillations^[^ ^42^ ^]^ induced by mitochondrial damage may also lead to mitotic catastrophe and cell death. Considering that mitochondrial damage is highly associated with immune system activation based on the current study,^[^ ^38^ ^]^ our work partially suggests the underlying anti‐tumor mechanisms by which CAP treatment induces immunogenic cell death.

Considering the importance of cellular metabolism and the notable mitochondrial dysfunction induced by CAP treatment, mitochondrial metabolism may be a therapeutic target for the clinical application of CAP. Several studies have focused on the inhibitory effects of CAP on the cellular energy metabolism. Tomoyuki et al. quantitatively clarified that CAP affected the final fate of tumor cells by controlling mitochondrial redox homeostasis and energy metabolism through biochemical modeling and numerical simulations.^[^ ^43^ ^]^ Guo et al. found that CAP treatment significantly reduced the expression of several key enzymes involved in glycolysis and inhibited glycolysis in malignant tumor cells.^[^ ^44^ ^]^ Tan et al. also confirmed that CAP treatment decreased the glycolysis level in hepatocellular carcinoma cells, activated the AKT/mTOR/HIF‐1α pathway, and finally induced apoptosis and autophagy.^[^ ^45^ ^]^ Kong et al. analyzed the metabolite profile of myeloma tumor cells treated with CAP with gas‐chromatography time‐of‐flight mass‐spectrometry, and demonstrated that CAP treatment significantly changed the metabolite profile of myeloma tumor cells, especially the change in the β‐alanine metabolic pathway.^[^ ^46^ ^]^ Our results also indicated that severe mitochondria dysfunction and metabolism disturbances, including the decreased glycolysis, oxidative phosphorylation, and ATP production, were instantly induced by low‐dose CAP treatment (Figure 6). Hence, our study suggests that mitochondrial metabolism is a potential therapeutic target for the clinical application of CAP.

## 4 Conclusion

**Figure 9:**
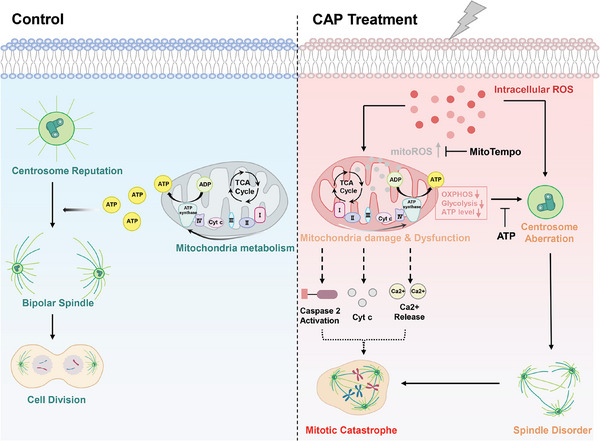
Schematic overview of the mechanism underlying CAP treatment inducing mitotic catastrophe in tumor cells.

We reported that CAP‐derived ROS radicals cause damage and dysfunction of mitochondria and subsequently induce mitotic catastrophe by suppressing microtubule dynamics and mitotic spindle assembly. Increased mitoROS and decreased ATP production are pivotal causes of mitotic defects (**Figure** **9**). We anticipate that our work will promote a better understanding of CAP treatment for solid tumors in vivo, improve CAP clinical application, and develop more therapeutic strategies.

## 5 Experimental Section

### CAP Equipment and Treatment

The atmospheric pressure dielectric barrier discharge (DBD) and jet plasma were both used in this study (Extended Figure 3). For the DBD plasma device, the CAP generator consisted of a hollow plexiglass cylinder as the reaction chamber with four electrodes and two orifices as gas inlet and outlet. The high‐voltage electrode is a 28 mm diameter copper cylinder with 1 mm thick quartz glass as the insulating dielectric barrier. The ground electrode was a 37 mm diameter copper cylinder, and the discharge gap between the bottom of the quartz glass and the surface of the medium was maintained at 5 mm. CAP was generated by a voltage of 12 kV (peak to peak) with a frequency of 24 kHz and helium gas (99.99%) was used as the working gas with a flow rate of 120 L h^−1^. Cells were seeded in 35‐mm‐diameter petri dishes with 1.5 mL complete medium in and then exposed directly to CAP for indicated time. Considering that CAP treatment exhibited the biological effects through reactive species, this study used a constant discharge power and lengthened the treatment time to increase the content of reactive species in different CAP treatment groups. To better describe the dose of CAP, the preset time was used to visually define the actual treatment dose of the CAP treatment.

As regards the jet device, a commercial atmospheric pressure plasma jet (CHNT, TDGC2‐2, Jiaxing, China) was used to treat the tumor‐bearing mice. The copper rod (diameter of 2 mm and total length of 100 mm) was mounted with one side sealed as a single electrode in the center of a quartz capillary (inner diameter of 2 mm, outer diameter of 4 mm, and length of 80 mm) and tightly wrapped in a polytetrafluoroethylene shell. As the nozzle, a small quartz tube (inner diameter of 4 mm, and outer diameter of 6 mm, 20 mm long) was connected to the shell. The operating conditions of the jet plasma included helium gas flow rates (400 L h^−1^) and generated by voltage of 12 kV (peak to peak) at a frequency of 24 kHz. The tumor xenografts bearing in mice were treated by jet plasma for 15 min one by one. The distance between the open end of quartz tube and the surface of xenografts was fixed at 2 cm during plasma treatment.

### Cell Culture and Reagents

The human lung cancer cell lines, Calu‐1 and H1299, were purchased from American Type Culture Collection (Manassas, USA). The other cell lines, human gastric cancer (MGC‐803 and MKN‐28), human colon cancer (HCT116 and SW480) and human renal clear cell carcinoma (786‐0 and 769‐P), were obtained from the Cell Bank of Type Culture Collection of Chinese Academy of Sciences (Shanghai, China). HCT116 and SW480 cells were cultured in high glucose DMEM (Hyclone, Logan, USA) supplemented with 10% (v/v) fetal bovine serum (FBS, Thermo Scientific Hyclone, Logan, USA) and 1% (v/v) penicillin/streptomycin (Gibco, Carlsbad, CA, USA). Other cells were cultured in RPMI 1640 (Hyclone, Logan, USA) supplemented with 10% (v/v) FBS and 1% (v/v) penicillin/streptomycin. All cells were maintained at 37 °C in a humidified incubator with 5% CO_2_ and 95% air.

Nocodazole was obtained from MedChem Express (Shanghai, China) and the propidium iodide (PI) was purchased from Sangon Biotech (Shanghai, China).

### Cell Death Analysis

Cells were solution stained with PI (5 µg mL^−1^) for 20 min at room temperature and then assessed with a flow cytometer (Accuri C6, BD Biosciences, Bedford, MA, USA). All the data analysis was performed with Flowjo analysis software (TreeStar, Ashland, OR, USA).

### Quantitative RT‐PCR

Total DNA was extracted with HiPure Total DNA Mini Kit (Magen, Guangzhou, China) following the manufacturer's instructions. Quantitative PCR reaction mixtures were prepared with Hieff qPCR SYBR Green Master Mix (Yeasen Biotechnology, Shanghai, China). PCR reactions were performed and analyzed on LightCycler 480 Instrument (Roche, Indianapolis, IN). PCR primers were shown as follows: 5′‐TTCTAATCGCAATGGCATTCCT‐3′, 5′‐AAGGGTTGTAGTAGCCCGTAG‐3′ (MT‐ND1); 5′‐ACACAACTGTGTTCACTAGC‐3′, 5′‐CAACTTCATCCACGTTCACC‐3′ (β‐globin); 5′‐CGGAACCGCTCATTGCC‐3′, 5′‐ACCCACACTGTGCCCATCTA‐3′ (GAPDH). Relative DNA levels were normalized to GAPDH level. The fold changes of mRNA were calculated with the 2^−ΔΔCt^ method.

### Detection of Cell Proliferation

Cell proliferation was detected with BeyoClick EdU Cell Proliferation Kit with Alexa Fluor 488 (Beyotime Biotechnology). After incubated with EdU (10 µm) for 2 h, the cells were fixed with 4% paraformaldehyde for 30 min, permeabilized with 0.3% Triton X‐100 for 15 min, and reacted with click additive working buffer for 30 min. The nuclei of cells were stained with Hoechst 33342 (10 µm, Sigma‐Aldrich, New York, USA) for 20 min, and then cells were photographed with a fluorescent microscope (Leica DMI 4000B, Germany). All images were collected with the same instrument parameters and processed with the same settings.

### Detection of Nuclei Morphology

After fixed, the cells were immediately stained with acridine orange (100 µg mL^−1^, Sigma‐Aldrich, New York, USA) for 3–5 min. After a thorough wash, cells were visualized with a fluorescent microscope (Leica DMI 4000B).

### Immunoblotting

Cells were collected and lysed with RIPA buffer supplemented with PMSF (Beyotime Biotechnology). The protein concentration was determined with BCA Protein Assay Reagent Kit (Beyotime Biotechnology). Approximately 40 µg total protein of each sample was separated by SDS‐PAGE and transferred onto PVDF membranes (Merck Millipore, Darmstadt, Germany). Sequentially, the membranes were blocked with 5% (w/v) defatted milk (BD/Difco, Sparks, MD, USA) for 1 h at room temperature followed by incubation with primary antibodies overnight at 4 °C. The primary antibodies against AMPK (1:1000, 5831S), phospho‐AMPKα (Thr172) (1:1000, 50081S), acly‐α‐tubulin (1:1000, 5335), Aurora A (1:1000, 14475S), phospho‐Aurora A (Thr288) (1:1000, 3079S) from Cell Signaling Technology, α‐tubulin from Proteintech (1:1000, 11224‐1‐AP) and β‐actin from HuaAn biotechnology (1:2000, EM21002) were used. After washing with TBST (0.1% Tween‐20 in Tris‐HCl buffer), the membranes were incubated with the secondary antibodies, IRDye‐labeled goat anti‐mouse IgG and anti‐rabbit IgG, (1:10000, LI‐COR Biosciences) for 1 h and imaged with Odyssey CLx Infrared Imaging system (Li‐COR Biosciences).

### Immunofluorescence

Cells were fixed with 4% (v/v) paraformaldehyde for 30 min, permeabilized and blocked with TNBS (0.2% Tritonx‐100 and 1% FBS in PBS) for 30 min. After that, the cells were incubated for 2 h with specific primary antibodies for different proteins as follows: anti‐α‐tubulin (1:200, 11224‐1‐AP, Proteintech), anti‐Aurora A (1:200, 9202S, Cell Signaling Technology) at 37 °C. After rinsing with TNBS solution, the cells were incubated for 1 h with Goat Anti‐Rabbit IgG H&L Highly Cross‐Adsorbed Secondary Antibody (1:1000, A11034, Invitrogen) at 37 °C. Hoechst 33342 (10 µg mL^−1^) was used to stain the nuclei. The cells were visualized with a fluorescent microscope (DMI4000B, Leica). All images were collected with the same instrument parameters and processed with the same settings. The number of cells with Aurora A foci in at least 200 cells was counted in each sample.

### Cell Cycle Analysis

The fixed cells were incubated with RNase A for 1 h (Beyotime Biotechnology) and stained with PI (2 µg mL^−1^) for 30 min at room temperature. After washing with prechilled PBS, the cells were immediately analyzed with a flow cytometer (Accuri C6, BD Biosciences). All the data analysis was performed with Flowjo analysis software (TreeStar, Ashland).

### Confocal Microscopy of Mitochondria

At 24 h after CAP treatment, the cells were incubated with Mitotracker Red (5 µm, Sigma‐Aldrich, New York, USA) for 30 min. Confocal images were obtained with a Zeiss LSM 780 confocal microscope.

### Cellular Bioenergetics Analysis

The mitochondrial energy activity was measured with a Seahorse Bioscience analyser (XF24, Seahorse Biosciences.) and the Seahorse XFp Cell Energy Phenotype Test Kit (Agilent) according to the manufacturer's instructions. Seahorse Wave software (v2.6) was applied for data processing and the final reports for the OCR and ECAR were generated by the Seahorse XF Cell Energy Phenotype report generator.

### Measurement of Mitochondrial Respiratory Chain Complex Activity

The activities of mitochondrial respiratory chain complexes, including complex I to V, were detected with the commercial reagent kits from Solarbio Life Science (Beijing, China). In brief, cells were collected, lysed with ultrasonication and resuspended with various detection solutions from these kits, respectively. After centrifuge at 12000× g for 10 min, the absorbance of supernatant was measured with a Varioskan Flash microplate reader (Thermo Fisher Scientific, Illinois, USA) and the analysis was performed following the manufacturer's instructions.

### Detection of Total ROS and Mitochondrial ROS

Total ROS and mitochondrial ROS were measured with the fluorescent probe H2‐DCFHDA (Beyotime Biotechnology) and MitoSOX Red (Invitrogen, Grand Island, NY, USA), respectively. After CAP treatment, the cells were incubated with fluorescent probes (10 µm for H2‐DCFHDA, 5 µm for MitoSOX Red) for 30 min in the cell incubator. Finally, the cells were analyzed with a flow cytometer (Accuri C6, BD Biosciences) and the data analysis was performed with Flowjo analysis software (TreeStar, Ashland).

### Measurement of ATP

Intracellular ATP level was measured with Enhanced ATP Assay Kit (Beyotime Biotechnology). The cells were lysed with ATP lysis buffer and centrifuged at 12000×g (4 °C, 5 min). The supernatant was harvested to react with ATP detection solution, and the fluorescence was read with a chemiluminescence apparatus (Turner Biosystems, Sunnyvale, CA, USA).

### Mitochondrial Membrane Potential Analysis

Mitochondrial Membrane Potential Assay Kit with JC‐1 (Beyotime Biotechnology) was used to assess the mitochondrial transmembrane potential according to the manufacturer's instructions. Briefly, the cells were stained with JC‐1 for 30 min and immediately analyzed with a flow cytometer (Accuri C6, BD Biosciences). All the data analysis was performed with Flowjo analysis software (TreeStar, Ashland).

### Microtubule Regrowth Assay

For detecting the influence of CAP treatment on microtubule polymerization, Nocodazole, a rapidly‐reversible inhibitor of tubulin, was exerted to depolymerize the microtubules. After CAP treatment, the cells were incubated with nocodazoleM (3 µm, MedChem Express) at 37 °C for 1 h and washed with pre‐warmed PBS buffer for three times. Subsequently, the cells were incubated in pre‐warmed fresh medium at 37 °C for 90 s to make microtubules regrow. After incubation, cell samples were fixed with 4% (v/v) paraformaldehyde and the microtubule regrowth was detected by immunofluorescence.

### Nude Mice Xenograft Models

Six‐week‐old male nude mice were obtained from GemPharmatech Company (Nanjing, China). Calu‐1 cells were suspended in prechilled PBS and 1 × 10^7^ cells were injected into the dorsal flank of nude mice subcutaneously. When the tumor volume reached 50–100 mm^3^, the mice were randomly divided into two groups (5 mice per group), namely, the control and the plasma‐treatment groups. To investigate whether the treatment effect of plasma was attenuated with increasing depth in solid tumor, tumor‐bearing mice were treated with jet plasma for 15 min every 2 days. After 20 days, mice were sacrificed, and the inoculated subcutaneous tumors were removed. The tumor volume was calculated as volume = length × width^2^ × 1/2. All animal experiments were approved by Hefei Institutes of Physical Science Experimental Animal Ethics Committee.

### TUNEL and IHC Analysis

For the TUNEL assay, a commercial kit (KeyGEN bioTech, Jiangsu, China) was used according to the manufacturer's instructions and cell death levels were quantified. Briefly, the sections of tumors were de‐paraffinized with xylene and ethanol. After being repaired with citrate buffer (pH 6.0) and incubated in boiling water for 20 min, the sections were detected with the commercial kit.

IHC, also called immunohistochemistry, is the combination of histology and immunology. The resulting technique is a powerful tool that allows researchers to not only determine whether particular antigens are present within a given cell but also identify the microanatomical (cellular) location of the antigen. For IHC analysis, tissue sections were incubated with anti‐4‐Hydroxynonenal (4‐HNE) antibody (0.1 µg mL^−1^; MAB3249, R&D Systems) at room temperature followed by incubation with HRP‐conjugated goat anti‐mouse IgG H&L (1:2000; ab6708, Abcam). After incubation with streptavidin peroxidase, sections were visualized with DAB substrate (Beyotime Biotechnology, Shanghai, China). All images were captured with Pannoramic Scan (3DHISTECH Ltd., Budapest, Hungary).

For the quantification of 4‐HNE and TUNEL, the digital pathology software 3DHISTECH's Slide Converter was used and the positive rates of 4‐HNE or TUNEL were determined at different depth levels based on setting the tumor tissue surface as the starting point. Positive staining was quantified with ImageJ software and the ratio was calculated as Positive area / Total area × 100%.

### Statistical Analysis

Statistical differences were assessed with GraphPad Prism 8.0 software (San Diego, CA, USA). Data represent mean ± SD from three independent experiments. P‐values were analyzed with Student's *t*‐test. *p *< 0.05 was considered significant and n.s. represented no significance.

## Conflict of Interest

The authors declare no conflict of interest.

## Supporting information



Supporting Information

## Data Availability

The data that support the findings of this study are available from the corresponding author upon reasonable request.
