# Supplementary material for: Unleashing the Power of Cold Atmospheric Plasma: Inducing Mitochondria Damage‐Mediated Mitotic Catastrophe
Source: Adv Sci (Weinh). 2024 Oct 23;11(46):2401842. doi: 10.1002/advs.202401842 (PMC11633534; doi:10.1002/advs.202401842)
Supplement: Supplementary file 1 — Supporting Information [file ADVS-11-2401842-s001.docx]

**Supplementary data**

**Abbreviation**

**CAP** Cold atmospheric plasma

**PAM** Plasma-activated medium

**ROS** Reactive Oxygen Species

**RNS** Reactive Nitrogen Species

**4-HNE** 4-Hydroxynonenal

**PI** Propidium Iodide

**DEGs** Differential expressed genes

**GO enrichment** Gene Ontology enrichment

**GSEA** Gene Set Enrichment Analysis

**GSVA** Gene Set Variation Analysis

**OCR** O2 consumption rate

**ECAR** Extracellular acidification rate

**mtDNA** Mitochondrial DNA

**mitoROS**  Mitochondrial ROS

**H&E stain** Hematoxylin and eosin stain

**IHC**  Immunohistochemistry


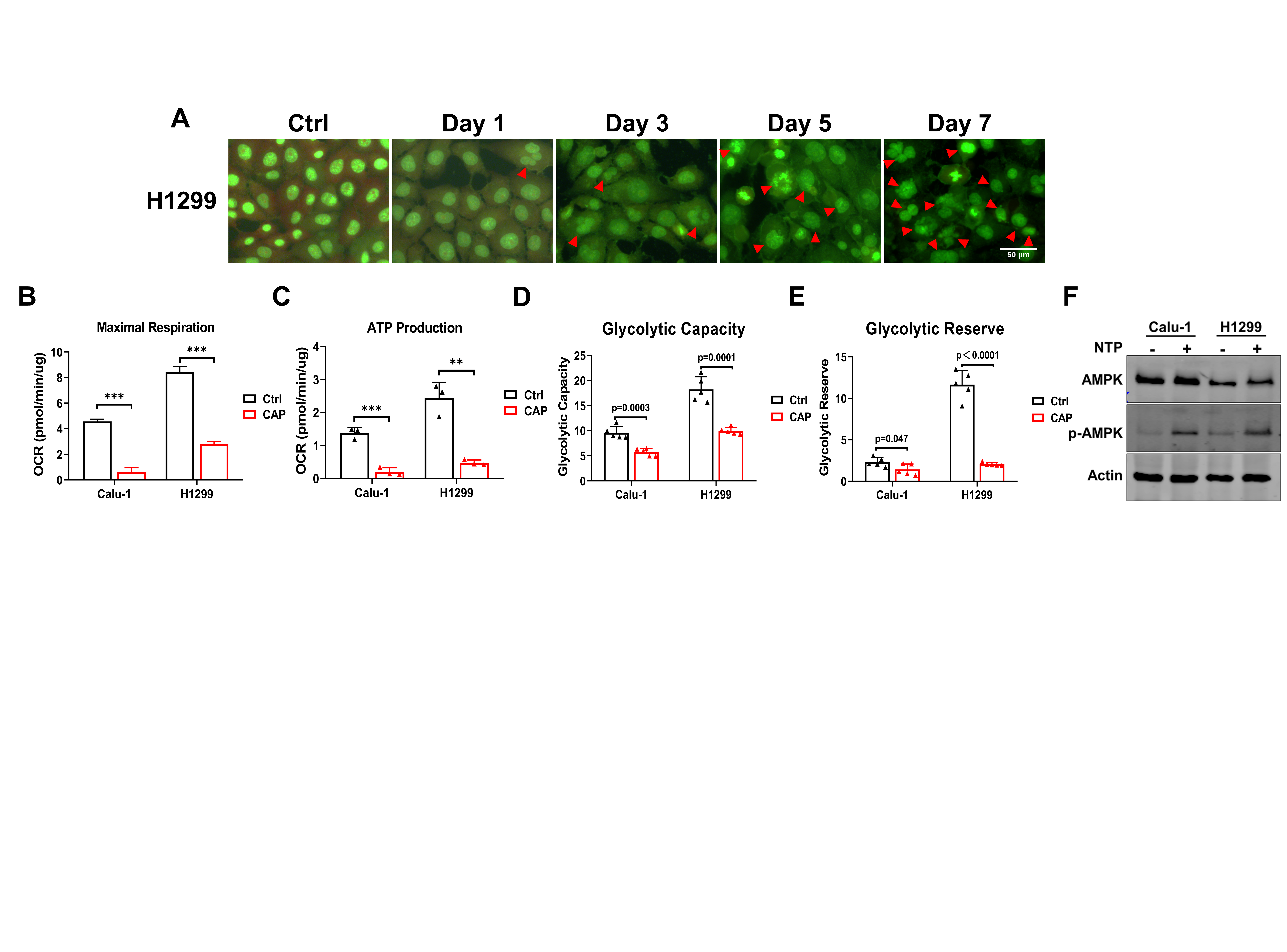


**Extended Figure 1. CAP treatment (20 s)** **induced mitochondrial damage. A.** Nucleus morphology of treated H1299 cells captured at indicated timepoints. Scale bar, 50 μm. **B.** The maximal respiration levels of Calu-1 and H1299 cells. **C.** The ATP production levels of Calu-1 and H1299 cells. **D.** The glycolytic capacity levels of Calu-1 and H1299 cells. **E.** The glycolytic reserve levels of Calu-1 and H1299 cells. **F.** The phosphorylation level of AMPK signaling pathway.

**
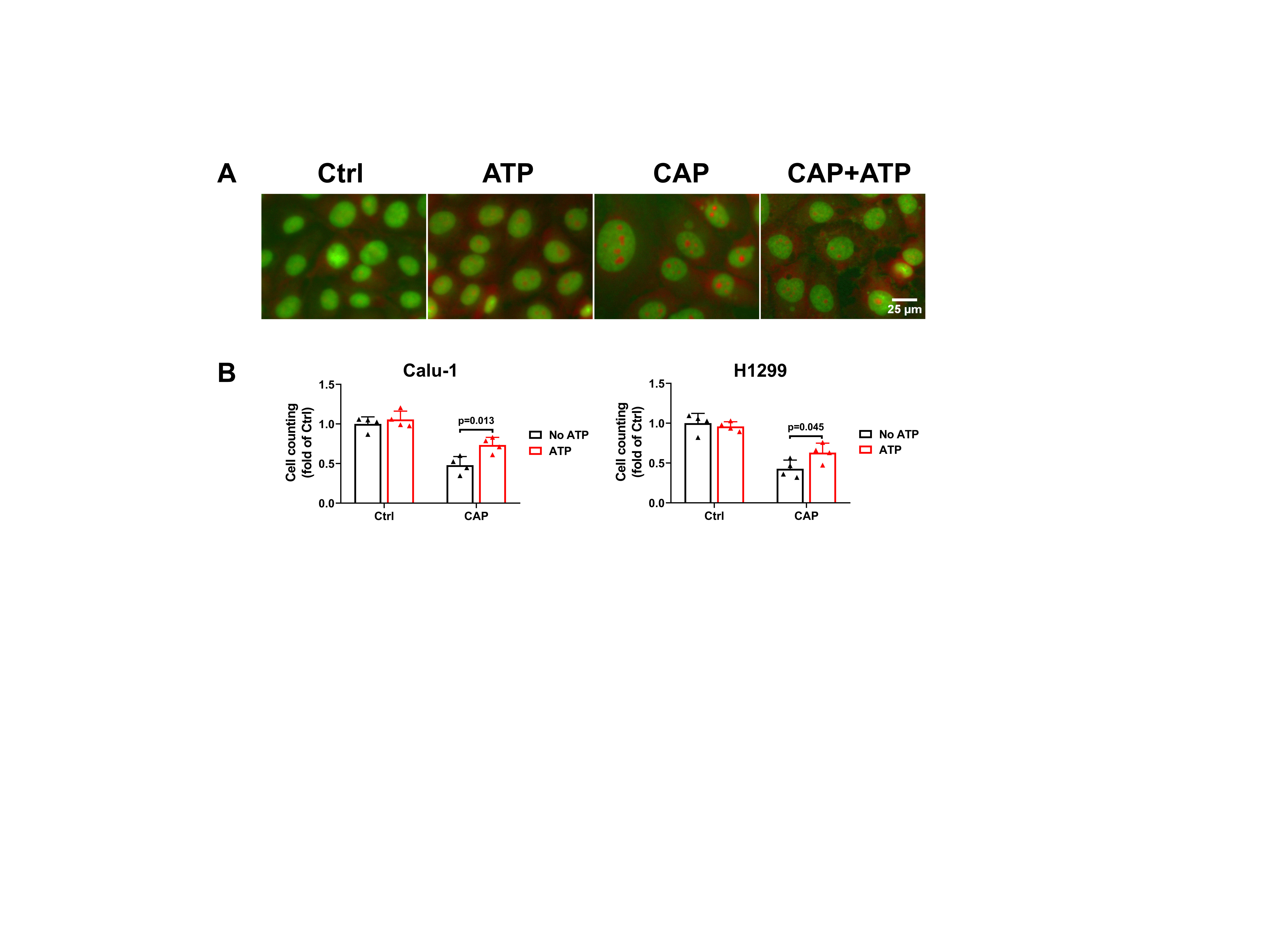
**

**Extended Figure 2. Exogenous ATP** **suppressed mitotic catastrophe and promoted proliferation. A**. Representative immunofluorescent images of nuclei (green) in H1299 cells at 72 h after CAP treatment with or without ATP (100 μM). Scale bar, 25 μm. **B.** The number of cells at 72 h after CAP treatment with or without ATP (100 μM).


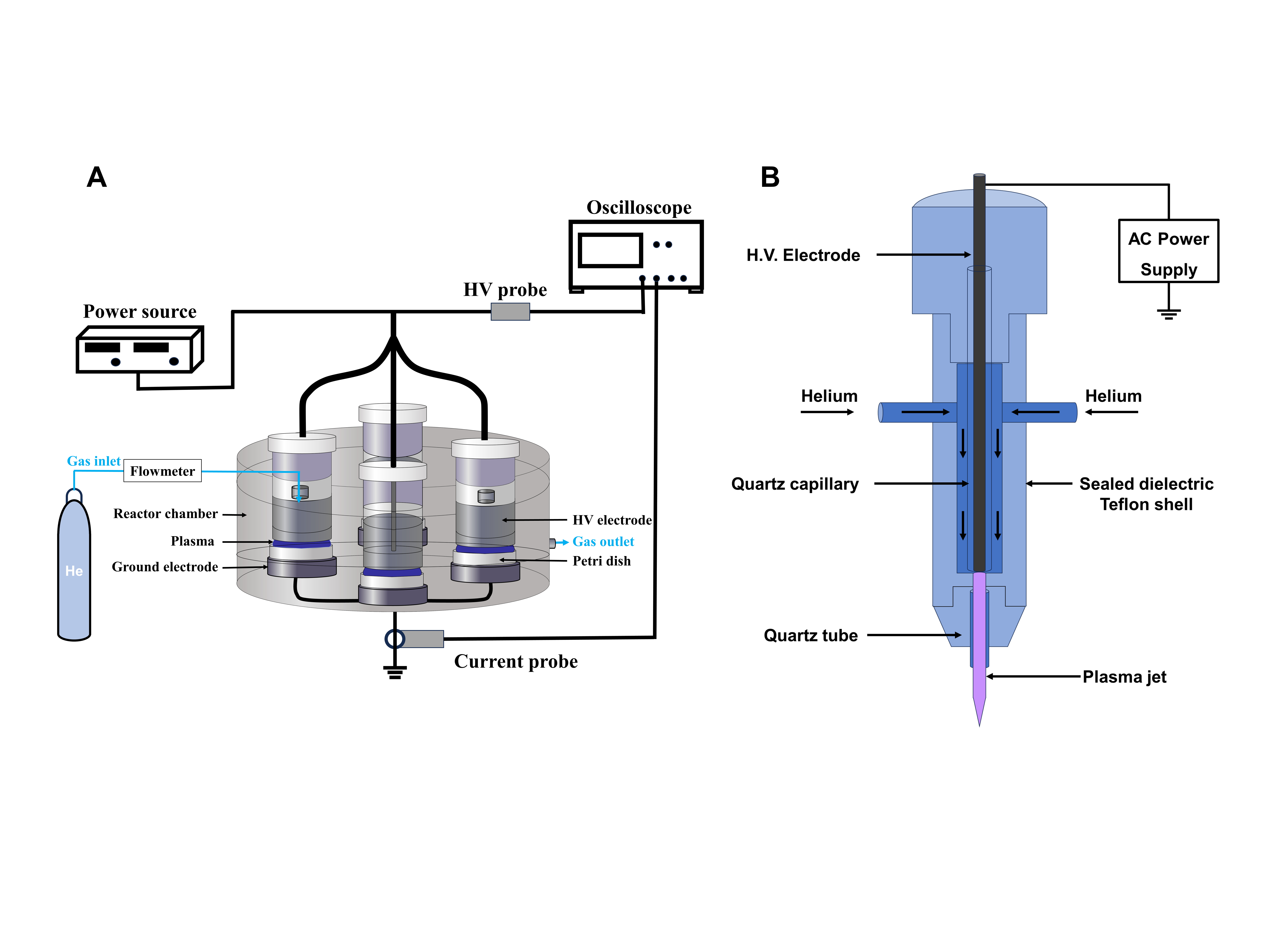


**Extended Figure 3. The schematic of the plasma devices. A.** The DBD plasma device. **B.** The jet plasma device.
